# Investigation of the relationship between subcarinal angle values and mortality in chronic obstructive pulmonary disease (COPD) patients in the intensive care unit

**DOI:** 10.3389/fmed.2026.1767952

**Published:** 2026-04-15

**Authors:** Ayşe Şahin Tutak, Hüseyin Avni Fındıklı, Ercan Çil

**Affiliations:** 1 Department of Internal Medicine, Adıyaman University School of Medicine, Adıyaman, Türkiye; 2 Department of Internal Medicine, Kahramanmaraş Necip Fazıl City Hospital, Kahramanmaraş, Türkiye; 3 Department of Chest Disease, Adıyaman University School of Medicine, Adıyaman, Türkiye

**Keywords:** COPD (chronic obstructive pulmonary disease), intensive care unit, mortality, PA-CXRs, SCA (subcarinal angle)

## Abstract

**Purpose:**

We aimed to investigate the relationship between subcarinal angle (SCA) values and mortality in patients with chronic obstructive pulmonary disease (COPD) patients admitted to the intensive care unit (ICU).

**Materials and methods:**

This retrospective study included 108 patients with COPD who were admitted to the ICU between January 2018 and December 2018. SCA values on posterior–anterior chest X-rays (PA-CXRs) were recorded from the patients’ Picture Archiving and Communication System (PACS). Patients were divided into survivor and non-survivor groups.

**Results:**

In this study, congestive heart failure was found to be associated with mortality (*p* = 0.011). In addition, higher creatinine levels (*p* = 0.034), elevated C-reactive protein (CRP) levels (*p* = 0.01), hypoalbuminemia (*p* = 0.018), narrower SCA values (*p* = 0.025), and higher Acute Physiology and Chronic Health Evaluation II (APACHE-II) scores (*p* = 0.001) were associated with mortality.

**Conclusion:**

Narrower SCA values, advanced age, elevated creatinine and CRP levels, hypoalbuminemia, and higher APACHE-II scores were associated with increased mortality in chronic obstructive pulmonary disease (COPD) patients admitted to the ICU. These findings suggest that SCA measured on routine chest radiographs may serve as a simple and readily available radiographic marker for mortality risk assessment in critically ill COPD patients.

## Introduction

The trachea is the major organ responsible for ventilation (1, 2). At the level of the fifth thoracic vertebra on the posterior–anterior chest X-ray (PA-CXR), the tracheal angles between the right and left main bronchi can be clearly observed (3). As observed on PA-CXRs, the angle formed between the right and left main bronchi at the tracheal bifurcation (carina) is referred to as the subcarinal angle (SCA) or bifurcation angle. Anatomically, a wide angle between the right main bronchus and the trachea indicates a narrow SCA. With aging, the right main bronchus moves closer to the midline, and the right bronchus almost becomes a continuation of the trachea (4). It is well established that tracheobronchial angles may vary depending on age, sex, inspiration and expiration, thoracic height and width, and sitting and lying positions. Obesity, left atrial enlargement, and thoracic surgery are associated with changes in tracheobronchial angles. Moreover, tracheobronchial angles may vary between two consecutive chest X-rays in the same individual (5–9). Although the SCA has anatomical and clinical relevance, a universally accepted physiologically “ideal” value has not been clearly determined. Previous studies have instead reported a relatively broad normal range for SCA measurements, indicating that this angle may vary depending on factors such as age, sex, body habitus, respiratory phase, and imaging technique (10–12).

Chronic obstructive pulmonary disease (COPD) patients is a chronic disease with high mortality. Intensive care unit (ICU) hospitalizations usually occur because of infections that exacerbate COPD attacks. In addition, exacerbations of comorbid conditions are among the other common reasons for frequent ICU hospitalizations. It is well known that the presence of one or more concomitant conditions in addition to COPD among COPD patients is an independent predictor of mortality regardless of whether such comorbid conditions are directly related to COPD (13, 14).

It is known that degeneration, vascularization, and ossifications may develop in the mucosal and cartilaginous tissues of the trachea in COPD patients (15–17). Such pathophysiological changes can lead to structural changes in the trachea, resulting in the narrowing of the tracheal lumen (18). As the diameter of the tracheal lumen changes in COPD patients, the anatomy of the trachea may be altered, resulting in a potential change in the bifurcation angle.

The accumulation of endotracheal secretions in the areas of degeneration in the trachea in COPD patients facilitates the emergence of clinical signs and symptoms associated with airway obstruction. Based on this hypothesis, we aimed to investigate the relationship between subcarinal angle values and mortality in patients with COPD admitted to the ICU. We did not find a study in the literature assessing the relationship between tracheobronchial angles and mortality in patients with COPD. Therefore, we believe that this study will make a meaningful contribution to the literature.

## Materials and methods

This retrospective single-center study was conducted in accordance with the ethical standards of the Declaration of Helsinki. Ethical approval was obtained from the Non-invasive Clinical Research Ethics Committee of Adıyaman University (approval date and number: 18 December 2018; 2018–9/2). The requirement for informed consent was waived by the ethics committee because the study was based on anonymized, routinely collected clinical data and involved no additional interventions.

**Figure 1:**
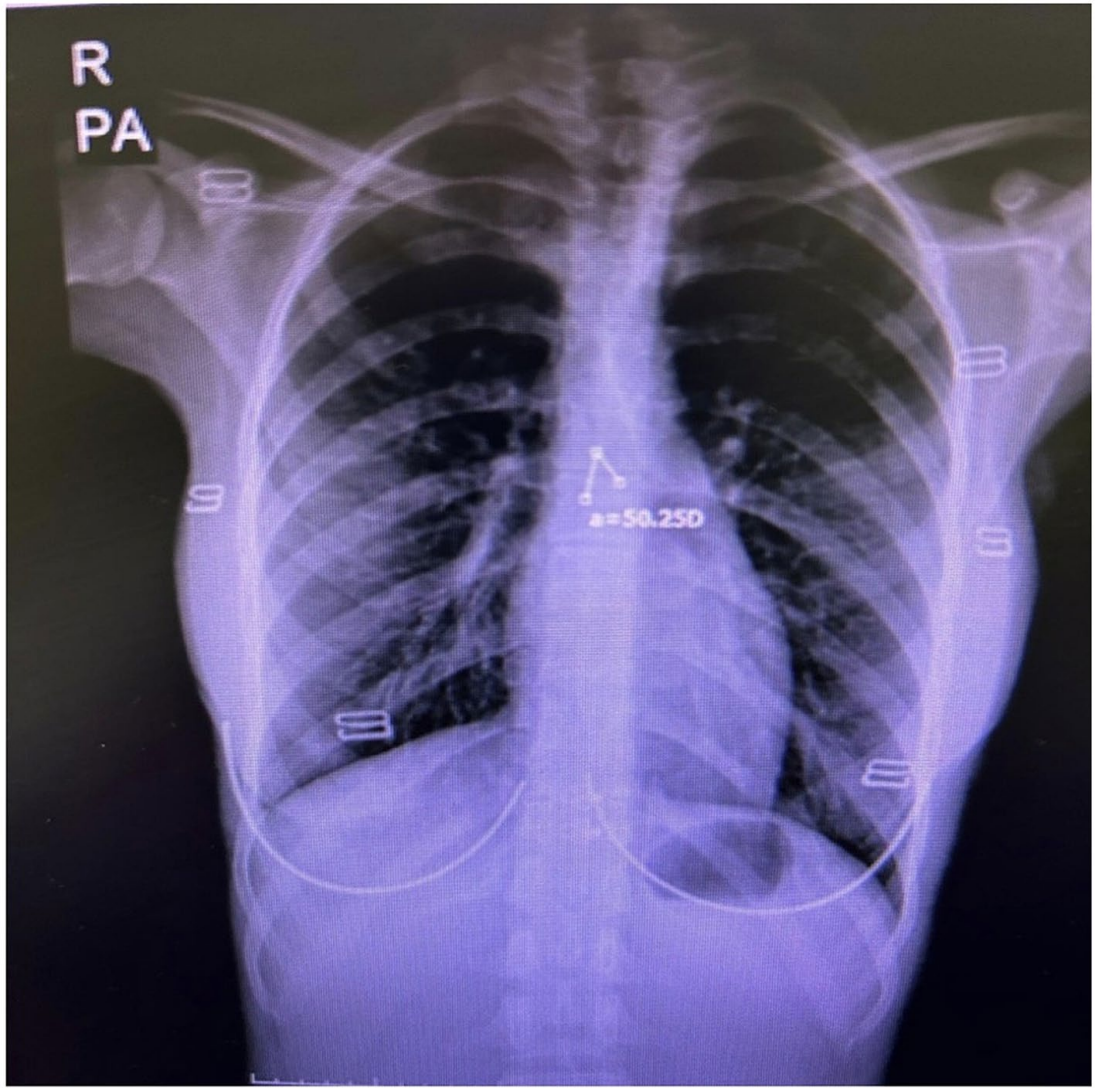
Measurement of the subcarinal angle (SCA) on a posteroanterior chest radiograph (PA-CXR) using the PACS. The subcarinal angle was measured on posteroanterior chest radiographs as the angle formed between the medial borders of the right and left main bronchi at the tracheal bifurcation. Measurements were taken using the digital measurement tools available in the PACS by evaluating the angle between the bronchial axes. Since there is no universally accepted threshold defining a “narrow” SCA in the literature, SCA values were analyzed as a continuous variable in the statistical analysis.

A total of 153 patients who were admitted to the tertiary-care ICU with a diagnosis of COPD were screened between January 2018 and December 2018. SCA values were calculated from admission posteroanterior chest X-rays using the Picture Archiving and Communication System (PACS) (Figure 1). APACHE-II scores, comorbidities, laboratory findings, and ICU discharge status (survivor or non-survivor) were obtained from the electronic medical records. A total of 45 patients were excluded due to missing clinical data or unavailable chest X-rays, and 108 patients were finally included in the analysis.

The inclusion criteria were as follows: patients aged 18–90 years, those diagnosed with COPD and admitted to the ICU, and those with available PA-CXRs, APACHE-II scores, and laboratory test results at the time of admission. The exclusion criteria included patients who had undergone prior chest wall surgery or mediastinal procedures, those with chest deformities, those with a history of infections causing fibrotic mediastinal or pulmonary changes, or those with a history of malignancy. A preliminary version of this study has been previously published as a preprint (19).

### Statistical analysis

Data analysis was performed using IBM SPSS Statistics version 24. The normality of the data was checked using the Shapiro–Wilk test. Continuous variables were expressed as mean ± standard deviation (SD) or median and interquartile range (IQR). Categorical variables were expressed as frequencies and percentages. The chi-squared test, Fisher’s exact test, the Mann–Whitney test, and Student’s t-test were used for data analysis as appropriate. A binary logistic regression analysis (enter method) was used to identify independent predictors of mortality of the study population. The receiver operating characteristic (ROC) curve and the area under the curve (AUC) with a 95% confidence interval (CI) were calculated for the SCA. The cutoff value, sensitivity, and specificity of the SCA for predicting mortality were also determined. All *p*-values of <0.05 were considered statistically significant.

## Results

The study included 108 patients, comprising 62 (57.4%) men with a mean age of 71.9 ± 13.4 years and 46 (42.6%) women with a mean age of 74.1 ± 13.1 years. The survivor group included 77 patients, and the non-survivor group included 31 patients. The mortality rate was determined to be 28.7%. The groups had similar characteristics in terms of sex distribution (*p* > 0.05). When the groups were analyzed in terms of age, the non-survivor group consisted of older individuals (*p* < 0.05). When the groups were examined in terms of comorbid conditions, congestive heart failure was more common in non-survivors than in survivors (*p* < 0.05). The groups were similar in terms of other comorbid conditions (*p* > 0.05). The median APACHE II (*p* < 0.01) score was higher in the non-survivor group, whereas the median SCA values (*p* < 0.05) were lower in the non-survivor group. The demographic and clinical characteristics of all study patients are shown in Table 1. According to the laboratory parameters, the mean values of creatinine and C-reactive protein (CRP) were significantly higher in the non-survivor group (*p* < 0.05). The mean value of albumin was higher in the survivor group (*p* < 0.05). The mean values of other laboratory parameters were similar in both groups. Laboratory characteristics of all patients included in the study are given in Table 2.

**Table 1:** Baseline characteristics of patients in the intensive care unit, stratified by survival status.

Characteristics	Total	Survivor	Nonsurvivor	*p*
	(𝑛 = 108)	(𝑛 = 77)	(𝑛 = 31)	
Age (years)	72.8 ± 13.2	71 ± 13.8	77.4 ± 10.5	0.022
Sex				0.440
Males	62 (57.4%)	46 (59.7%)	16 (51.6%)	
Females	46 (42.6%)	31 (40.3%)	15 (48.4%)	
Comorbidities				
COPD	108 (100%)	-	-	-
Hypertension	41 (38%)	27 (35.1%)	14 (45.2%)	0.328
Congestive heart failure	19 (17.6%)	9 (11.7%)	10 (32.3%)	0.011
Coronary artery disease	18 (16.7%)	14 (18.2%)	4 (12.9%)	0.505
Stroke	13 (12%)	6 (9.1%)	7 (19.4%)	0.125
Diabetes	26 (24.1%)	17 (22.1%)	9 (29%)	0.444
Renal disease	16 (14.8%)	9 (11.7%)	7 (22.6%)	0.229
Liver disease	12 (11.1%)	7 (9.1%)	5 (16.1%)	0.320
Others	15 (13.9%)	9 (11.7%)	6 (19.4%)	0.358
APACHE-II score	25 (20–32)	23 (18–29)	29 (22–44)	0.001
SCA (subcarinal angle)	81° (68°–92°)	85° (72°–92°)	73° (62°–84°)	0.025

**Table 2:** Comparison of laboratory parameters between survivors and non-survivors in the intensive care unit.

Parameters	Total	Survivor	Non-survivor	*p*-value
	(*n* = 108)	(*n* = 77)	(*n* = 31)	
Leukocytes	12.65 (8–16.6)	12 (8–15)	15.1 (8.1–17.6)	0.110
Hemoglobin (g/dL)	10.3 (8.5–12.25)	10.6 (8.5–12.4)	10 (8.4–11.8)	0.472
Thrombocytes (x 10^3^/μL)	199 (84–269)	204 (101–298)	160 (66–252)	0.095
Sodium (mg/L)	137 (133–140)	137 (133–140)	138 (132–140)	0.868
Potassium	4.1 (3.3–4.8)	3.9 (3.2–5)	4.4 (3.8–4.8)	0.089
Creatinine (mg/dL)	1.58 (0.81–2.43)	1.25 (0.81–2.19)	1.8 (0.97–3.5)	0.034
Albumin (g/dL)	2.685 (2.13–3.2)	2.88 (2.22–3.29)	2.44 (1.8–2.78)	0.018
C-reactive protein (mg/L)	38.4 (20.5–54)	35.9 (19–47.4)	46 (21.1–69)	0.010

**Figure 2:**
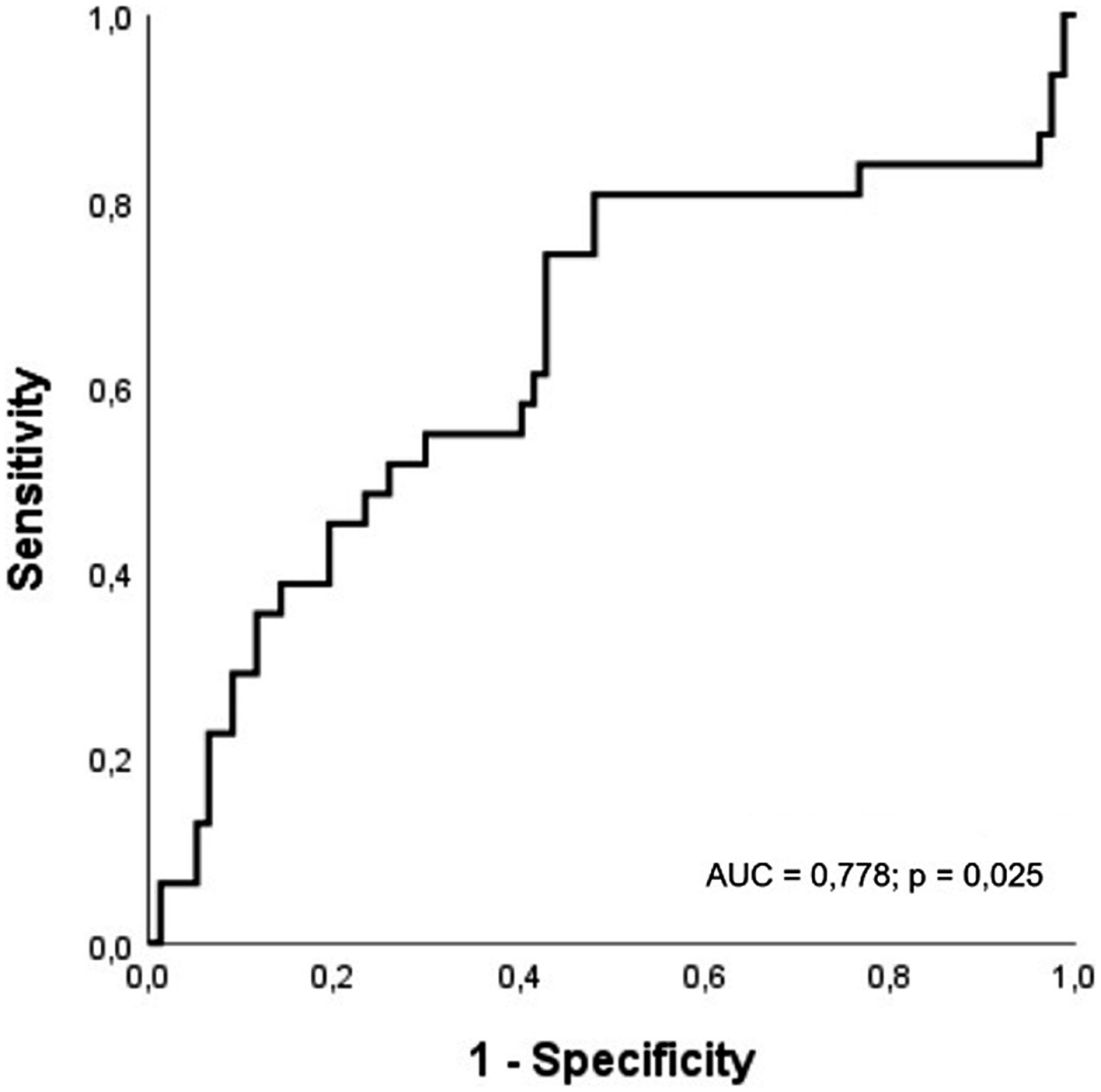
The receiver operating characteristic (ROC) curve illustrating the predictive value of the subcarinal angle (SCA) for intensive care unit mortality.

To determine the clinical and laboratory predictors of mortality in ICU patients, a logistic regression analysis was used. Age (OR = 1.073; 95% CI: 1.017, 1.132; *p* = 0.010), congestive heart failure (OR = 4.912; 95% CI: 1.266, 19.05; *p* = 0.021), APACHE-II score (OR = 1.109; 95% CI: 1.042, 1.179; *p* = 0.001), creatinine (OR = 1.432; 95% CI: 1.017, 2.016; *p* = 0.039), and SCA values (OR = 0.960; 95% CI: 0.925, 0.996; *p* = 0.032) were independently associated with ICU mortality (Table 3). As shown in Figure 2, the SCA had an area under the curve (AUC) of 0.778 (95% CI: 0.513–0.964; *p* = 0.025). A cutoff value of ≤ 79.8 yielded a sensitivity of 58.1% and a specificity of 58.4%.

**Table 3:** Multivariate logistic regression model for predicting mortality in ICU patients.

Parameters	B	Wald	*p*-value	OR	95% C.I.
Age (years)	0.070	6.611	0.010	1.073	1.017–1.132
APACHE-II score	0.103	10.621	0.001	1.109	1.042–1.179
C-reactive protein (mg/L)	0.021	2.696	0.101	1.021	0.996–1.046
Albumin (g/dL)	−0.591	2.004	0.157	0.554	0.245–1.255
Creatinine (mg/dL)	0.359	4.241	0.039	1.432	1.017–2.016
Congestive heart failure	1.592	5.296	0.021	4.912	1.266–19.05
SCA	−0.041	4.606	0.032	0.960	0.925–0.996
Constant	−6.399	4.957	0.026	0.002	

## Discussion

In this study, we investigated the relationship between subcarinal angle (SCA) values and mortality in patients with chronic obstructive pulmonary disease (COPD) patients admitted to the intensive care unit. Our findings demonstrated that SCA values were significantly associated with mortality. In addition, several clinical and laboratory parameters, including advanced age, congestive heart failure, elevated creatinine and CRP levels, hypoalbuminemia, and higher APACHE-II scores, were also associated with mortality. These findings suggest that SCA, which can be easily measured on routine chest radiographs, may have potential clinical relevance in the risk assessment of critically ill COPD patients.

Comorbid conditions are major parameters investigated for their value in predicting mortality in COPD patients. In particular, cardiovascular diseases are important factors affecting prognosis (20, 21).

These age-related anatomical shifts are clinically reflected in increased rates of aspiration pneumonia and atelectasis, particularly in the right lower and middle lobes, due to the accumulation of secretions in the airway lumens of immobilized patients. These changes, often compounded by tracheomalacia—which is common in advanced age and COPD—result in significant damage to cartilage tissue and alterations of bronchial angles, further contributing to higher mortality and morbidity rates (5–9).

It is known that SCA becomes narrower with increasing age (22). In our patient group, a narrow SCA was observed in patients older than 70 years. Particularly in non-survivors, the mean age was higher (*p* = 0.022), and the SCA was narrower (*p* = 0.025).

In one study (23), no significant differences were found in SCA values between groups with COPD and groups without COPD. However, another study reported that tracheobronchial angles were associated with COPD severity and the presence of emphysema (24). A study conducted in 2017 showed that with increasing COPD severity, as detected by pulmonary function tests (PFTs), the size and transverse diameter of the heart decreased while lung volume increased on CT imaging (25). Based on these observations, it is reasonable to predict that tracheobronchial angles will change as the COPD severity increases. It is also known that mortality increases as COPD becomes severe. In our study, we compared survivors and non-survivors and found that the SCA was narrower (73°) in non-survivors. Based on these observations and predictable mechanisms, it can be concluded that changes in tracheobronchial angles during breathing may be associated with COPD severity.

It is known that APACHE-II scores predict mortality in patients with COPD (26, 27). In our study, APACHE-II scores were found to be higher in non-survivors. The relationship between hypoalbuminemia and mortality in COPD patients has been well documented in many studies (28–30). In our study, hypoalbuminemia was significant in non-survivors (Table 2). Similarly, elevated CRP levels have been shown in many studies to be associated with mortality and morbidity in patients with COPD (31, 32). Consistent with these findings, our study found that elevated CRP levels were associated with mortality (Table 2).

Although SCA measurements were performed using two-dimensional (2D) posteroanterior chest radiographs, all images were obtained from the same radiology unit, using the same equipment and protocols, which helped minimize measurement variability. Nevertheless, the use of 3D imaging and respiratory phase–standardized protocols would be preferable in future studies to achieve a more accurate anatomical representation. This study included only patients with COPD. The absence of a comparison group consisting of healthy individuals or patients with other pulmonary conditions limits the ability to assess the specificity of the relationship between SCA and mortality. Additionally, although major comorbidities were analyzed and congestive heart failure was identified as an independent predictor of mortality, comprehensive scoring systems, such as the Charlson Comorbidity Index, were not employed. Future studies may benefit from incorporating such tools for more detailed adjustment of confounding variables. Furthermore, other potential anatomical or physiological factors that may influence subcarinal angle measurements, such as obesity, thoracic anatomical variations, or left atrial enlargement, were not systematically evaluated in the present study.

Despite these limitations, this study has several notable strengths. To the best of our knowledge, it is among the first studies to investigate the relationship between subcarinal angle measurements and mortality in COPD patients admitted to the intensive care unit. In addition, the use of routinely obtained chest radiographs makes this parameter easily applicable in clinical practice.

These findings may contribute to improved risk stratification and the early identification of high-risk COPD patients in critical care settings.

We conclude that a narrow SCA angle, advanced age, high APACHE-II scores, hypoalbuminemia, and elevated CRP levels are all associated with increased mortality in COPD patients. Although it is difficult to define a universal standard range for SCA values due to anatomical and methodological variability, we believe that establishing a standardized measurement approach in future studies may enhance the predictive utility of SCA in clinical practice.

## Data Availability

The original contributions presented in the study are included in the article/supplementary material, further inquiries can be directed to the corresponding author.
